# Hypertension Management Beyond Blood Pressure Control in India: An Expert Consensus on Angiotensin II Receptor Blocker and Calcium Channel Blocker Combination Therapy With Reference to Telmisartan and Cilnidipine

**DOI:** 10.7759/cureus.103320

**Published:** 2026-02-09

**Authors:** Jagdish Hiremath, Ashok Kirpalani, Deodatta Chafekar, P. B Jayagopal, Saumitra Ray, V. K Chopra, Sameer Dani, Kamlakar Tripathi, J. P. S Sawhney, Abraham Oomman, Sunil Sathe, Jabir Abdullakutty, Vijay Kher, Deepak Dewan, Kamal Sharma, Mukesh Shete, P. S Vali, Sanjay Jain, Onkar C Swami

**Affiliations:** 1 Cardiology, Ruby Hall Clinic, Pune, IND; 2 Nephrology, Bombay Hospital, Mumbai, IND; 3 Nephrology, Supreme Kidney Care, Nashik, IND; 4 Cardiology, Lakshmi Hospital, Palakkad, IND; 5 Cardiology, Manipal Hospital, Kolkata, IND; 6 Cardiology, Max Super Speciality Hospital, New Delhi, IND; 7 Cardiology, Apollo Cardiovascular Health and Fitness Heart Institute, Ahmedabad, IND; 8 Nephrology, Institute of Medical Sciences, Varanasi, IND; 9 Cardiology, Sir Ganga Ram Hospital, Delhi, IND; 10 Cardiology, Apollo Hospital, Chennai, IND; 11 Cardiology, N. M. Wadia Institute of Cardiology, Ruby Hall Clinic, Pune, IND; 12 Cardiology, Lisie Hospital, Kochi, IND; 13 Nephrology, Epitome Kidney Urology Institute and Lions Hospital, New Delhi, IND; 14 Nephrology, Regency Super Specialty Hospital, Lucknow, IND; 15 Cardiology, SAL Hospital and Medical Institute, Gujarat, IND; 16 Nephrology, Jupiter Hospital, Mumbai, IND; 17 Nephrology, Asian Institute of Urology and Nephrology, Hyderabad, IND; 18 Medical Services, Alembic Pharmaceuticals Ltd, Mumbai, IND

**Keywords:** cilnidipine, combination therapy, hypertension, telmisartan, type 2 diabetes mellitus

## Abstract

Hypertension is a leading cause of cardiovascular morbidity and mortality globally, with particularly low control rates in India. Beyond controlling blood pressure (BP), effective management must address associated comorbidities such as cardiovascular disease (CVD), chronic kidney disease (CKD), and type 2 diabetes mellitus (T2DM). A multidisciplinary panel of experts, including cardiologists, nephrologists, and physicians, developed this consensus through six advisory board meetings. A comprehensive literature review of randomized controlled trials and systematic reviews published between 2019 and 2025 was conducted via PubMed and Google Scholar. Key topics included pharmacological and non-pharmacological management of hypertension, combination therapy, and comorbidity-focused treatment strategies. Early screening for hypertension-mediated organ damage and the use of combination therapy, specifically telmisartan and cilnidipine for their complementary benefits, were emphasized. Evidence showed improved BP control, reduced proteinuria, and organ protection in individuals with CVD, CKD, and T2DM. Non-pharmacological interventions such as dietary modification, physical activity, smoking cessation, stress management, and adherence to therapy through fixed-dose combinations were also endorsed. This expert consensus supports a comprehensive, patient-centered approach to hypertension management in India. Available evidence suggests that cilnidipine and telmisartan may offer potential benefits beyond BP reduction, largely based on pharmacologic properties and surrogate outcomes. Future large-scale, randomized, comparative outcome trials are needed to better define the relative clinical benefits of this combination.

## Introduction and background

Hypertension, often referred to as a "silent killer," is a leading cause of cardiovascular disease (CVD) and mortality worldwide, with a particularly high burden in India. According to the World Health Organization (WHO), 1.28 billion adults aged 30 to 79 years are currently living with hypertension, yet only 42% have been diagnosed, and a mere 21% have their condition under control [1]. In India, hypertension is emerging as a significant public health challenge, driven by factors such as urbanization, shifting dietary patterns, and increasingly sedentary lifestyles. The Indian Council of Medical Research-India Diabetes (ICMR-INDIAB) study estimated the overall prevalence of hypertension at 35.5%, with a higher prevalence in men (38.7%) compared to women (32.6%) [2]. Additionally, the National Family Health Survey (NFHS-5) reports that 24% of men and 21% of women in India are affected by hypertension [3]. According to a systematic review and meta-analysis by Koya et al. (51 studies, n=338313), the pooled hypertension control rate in India was 17.5% (95% CI: 14.3-20.6%) [4]. 

High blood pressure (BP) plays a crucial role in the development of serious health issues, such as myocardial infarction, stroke, and heart failure (HF). Additionally, it is associated with a range of other conditions, including renal disorders, diabetes, obesity, obstructive sleep apnea (OSA), and fatty liver disease [5-9]. Current guidelines stress the need to screen individuals with elevated BP for signs of end-organ damage, known as hypertension-mediated organ damage (HMOD) [10-14]. Identifying HMOD is crucial in the diagnosis and follow-up of hypertension [15]. Major risk factors for developing hypertension include advancing age, family history, the presence of comorbidities like diabetes or kidney disease, a sedentary lifestyle, overweight or obesity, high intake of dietary salt and fat, low intake of fruits and vegetables, and the use of tobacco and alcohol [16]. These risk factors underscore the importance of addressing both hypertension and its associated comorbidities in a comprehensive management plan. 

The rising prevalence and burden of hypertension and its comorbidities, along with the inadequate control rates in many regions, highlight the urgent need for a clear and actionable approach to manage this condition effectively. This consensus statement seeks to provide a comprehensive overview of the benefits of managing hypertension beyond merely controlling BP, emphasizing the reduction of complications and improving overall cardiovascular health. In this context, “management beyond BP control” primarily refers to risk modification through effective BP lowering, prevention or attenuation of HMOD, and improvement in intermediate or surrogate markers (such as proteinuria, microalbuminuria, and 24-hour BP control), rather than direct evidence of reductions in hard clinical endpoints such as myocardial infarction, stroke, end-stage renal disease, or mortality for any specific drug combination. 

**Figure 1:**
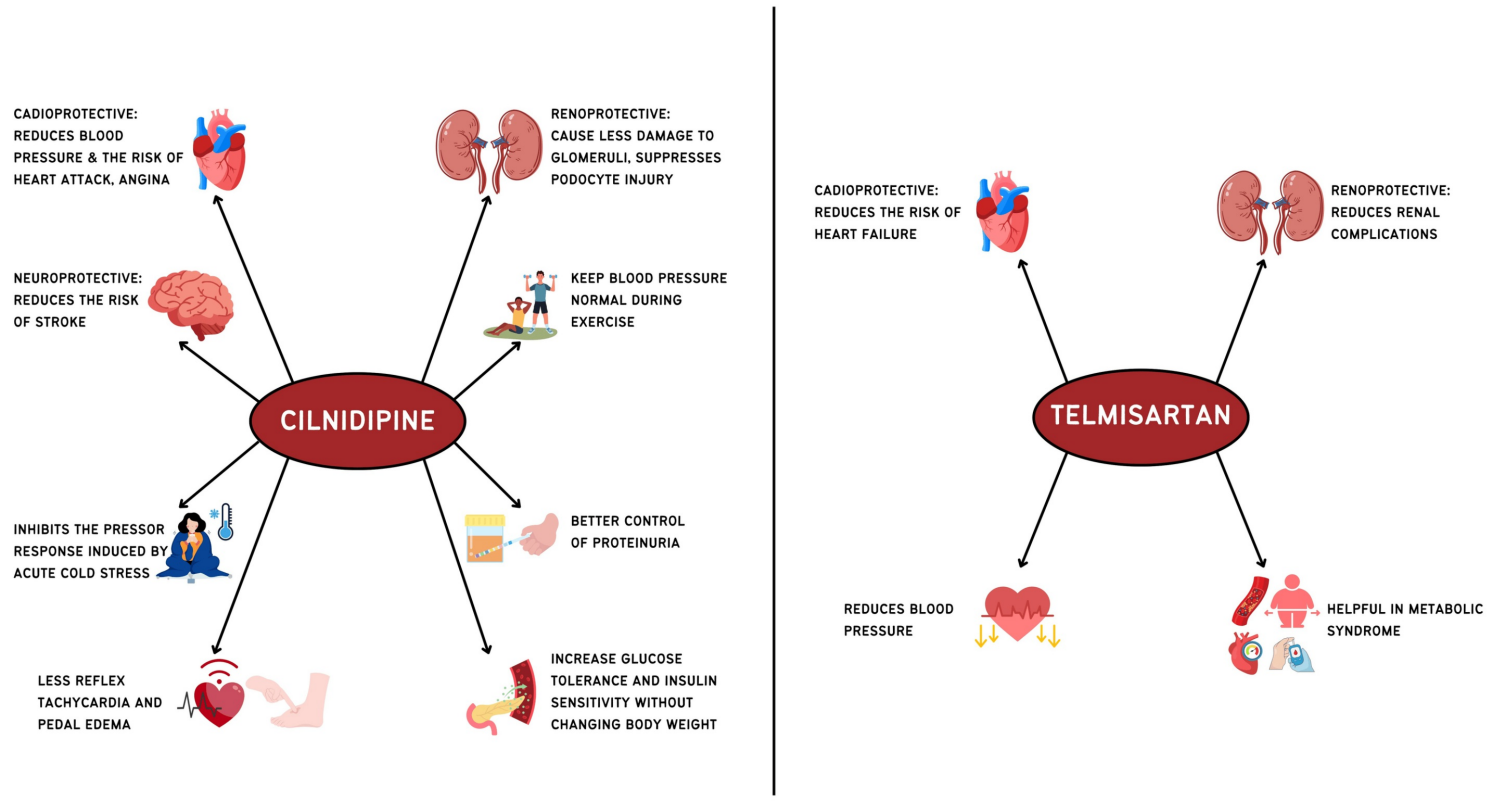
Pleiotropic benefits of cilnidipine and telmisartan Image credits: Authors

A central focus of this consensus is the role of antihypertensive drug classes, particularly angiotensin II receptor blockers (ARBs) and calcium channel blockers (CCBs), in the management of hypertension and its associated comorbidities. Telmisartan and cilnidipine are discussed as representative examples within the ARB and CCB classes, respectively, based on available evidence and clinical experience, and are not intended to imply exclusivity, preference, or superiority. Although direct clinical evidence evaluating the telmisartan-cilnidipine combination is limited, each agent has demonstrated established efficacy within its respective class. On this basis, the expert panel considers this combination to be a rational therapeutic option and has synthesized the available evidence to provide practical, context-specific guidance, while recognizing the need for future large, randomized, and multicenter studies to clarify comparative clinical outcomes. To ensure clarity of interpretation, this document distinguishes between established class-level effects and guideline-based recommendations for ARBs and CCBs; guideline-endorsed treatment strategies for hypertension in the presence of common comorbidities, and agent-specific observations derived largely from pharmacologic rationale, surrogate outcomes, or heterogeneous clinical studies. Any agent-specific discussion should therefore be interpreted as illustrative rather than preferential or outcome-definitive. Cilnidipine, a CCB, can block both L-type and N-type calcium channels. Telmisartan is an ARB specifically targeting the angiotensin II type 1 (AT1) receptor (Figure 1). These medications offer additional protective effects, particularly for individuals with cardiovascular and renal complications. 

## Review

Materials and methods 

This expert consensus was developed by a multidisciplinary panel of 57 healthcare professionals, including cardiologists, nephrologists, diabetologists, and internal medicine physicians, with extensive clinical experience in hypertension management across India. The objective was to formulate India-specific, practice-oriented recommendations for hypertension management beyond BP control, with emphasis on comorbidities, treatment adherence, reduction of pill burden, and real-world applicability. A structured literature search was conducted using PubMed and Google Scholar to identify relevant evidence published between January 2019 and March 2025, focusing on randomized controlled trials (RCTs), systematic reviews, and meta-analyses. Search terms included combinations of “hypertension,” “elevated blood pressure,” “angiotensin receptor blocker,” “ARB,” “telmisartan,” “calcium channel blocker,” “CCB,” “cilnidipine,” “combination therapy,” “monotherapy,” “systolic blood pressure,” “diastolic blood pressure,” “diet,” “physical activity,” “lifestyle modification,” “adherence,” and “fixed-dose combinations.” Filters were applied to include human studies, adult populations, and English-language publications.

Evidence selection prioritized studies addressing hypertension management, combination therapy, and hypertension-related comorbidities with relevance to clinical practice, including both surrogate and clinical outcomes, while excluding case reports, narrative reviews, editorials, opinion pieces, preclinical or animal studies, and studies with unclear methodology or insufficient outcome reporting. Given the nature of a consensus document, a formal PRISMA flow diagram and quantitative risk-of-bias scoring were not undertaken; instead, evidence was qualitatively synthesized based on study design, sample size, and clinical relevance to highlight established guideline-level recommendations and evidence gaps pertinent to Indian practice. Evidence summaries were prepared by a core writing group and circulated to all panel members in advance of six multidisciplinary advisory board meetings conducted across different regions of India. During these meetings, draft statements were presented and subjected to structured evidence review, open discussion, and sharing of real-world clinical experience, allowing for iterative refinement across multiple rounds. Divergent views were explicitly solicited and discussed; where disagreement persisted, statements were modified to reflect class-level recommendations, conditional language, or acknowledged uncertainty, and statements lacking broad agreement were revised or excluded. Following the advisory board meetings, revised drafts were circulated electronically, and outstanding questions or clarifications were addressed through subsequent online discussions, conducted either in small groups or one-to-one with panel members, to ensure alignment and resolve residual concerns prior to finalization. Consensus was defined as general expert agreement without sustained unresolved objection, rather than by formal Delphi methodology or predefined numerical voting thresholds, and this methodological approach and its limitations are explicitly acknowledged. All panel members disclosed potential conflicts of interest prior to participation. No organization had a role in evidence selection, data interpretation, formulation of recommendations, or final manuscript approval; editorial control and scientific content were determined exclusively by the expert panel.

Results and discussion

Management of Hypertension 

Hypertension is defined by the American College of Cardiology/American Heart Association (ACC/AHA) and Research Society for the Study of Diabetes in India (RSSDI) as a BP of 130/80 mm Hg or higher, while other guidelines set the threshold at 140/90 mm Hg or higher [10-14]. The primary goal of preventing and treating elevated BP and hypertension is to reduce CVD, enhance quality of life, and prevent premature death. Individuals with hypertension should be educated on the importance of regular BP monitoring, adhering to prescribed medications, and attending routine clinic visits to ensure effective management [17]. 

Non-pharmacological Management 

Individuals should receive guidance on adopting a healthy diet, staying physically active, losing weight, quitting smoking, managing stress, getting good sleep, and reducing alcohol and caffeine consumption. A systematic review and meta-analysis by Cernota et al. (23 studies, n=18153) on non-pharmacological interventions reported that educational strategies improved adherence and lifestyle modifications improved BP [18]. Refer to Table 1 for non-pharmacological management strategies [10-36]. Table 2 shows the consensus recommendation for non-pharmacological management. 

**Table 1:** Non-pharmacological management BMI: body mass index; CKD: chronic kidney disease; DASH: dietary approaches to stop hypertension; DBP: diastolic blood pressure; HIIT: high-intensity interval training; HR: hypertension risk; MAP: mean arterial pressure; RCT: randomized controlled trial; SBP: systolic blood pressure; WMD: weighted mean difference; OR: odds ratio; MNT: medical nutrition therapy

Management strategy	Characteristics/guideline recommendations	Evidence
Medical nutrition therapy and weight reduction	DASH: the diet should consist of whole grains, more vegetables (high in nitrates such as leafy vegetables and beetroot) and fruits, low-fat dairy products, poultry, fish, legumes, nuts, seeds, non‐tropical vegetable oils, and reducing intake of sweets, sugar‐sweetened beverages, and red meat [10,12-14]	A systematic review and meta-analysis by Jiao et al. (27 studies, n=7409) reported that various dietary models helped in reducing systolic (SBP: -WMD=-3.94, 95% CI (-5.10, -2.77), p<0.001) and DBP (WMD=-2.32, 95% CI (-3.01, -1.63), p<0.001) [19]
		Theodoridis et al. (12 studies) reported that adherence to the DASH diet reduced hypertension risk (HR: 0.81, 95% CI (0.73-0.90), OR: 0.80, 95% CI (0.70-0.91)) [20]
	Foods rich in magnesium, calcium, and potassium, and low in unsaturated and total fats, should be consumed	An RCT by Neal et al. reported that salt substitution (75% sodium chloride and 25% potassium chloride by mass) reduced the risk of stroke, major cardiovascular events, and death [21]
	The goal for sodium should be less than 1500 mg/day, and the aim should be less than 1000 mg/day. Avoid fast foods and processed foods. Low in saturated and total fats; limit salt intake to less than 5 g per day [10,12,13]	A systematic review and meta-analysis by Huang et al. (133 studies, n=12197) reported that a reduction in dietary sodium reduced SBP (-4.26 mm Hg) and DBP (2.07 mm Hg) [23]
	A diet rich in potassium (broccoli, tomatoes, soybeans, bananas, apricots, and potatoes), up to 3.5-5 g/day, is advised; in cases of advanced CKD, potassium intake should be reduced to <2.4 g/day [10,12,22]	Filippini et al. (32 studies) reported that adequate intake of potassium helps in lowering BP [24]
	Weight reduction (at least 1 kg) through exercise, low sodium intake, DASH and Mediterranean diet, and pharmacological management are recommended [10,12]	Yang et al. (35 studies, n=3219) reported that with an increase in the reduction of BMI, there was more reduction in SBP (-8.54 mm Hg (with BMI reduction ≥3 kg/m^2^)) and DBP (-3.45 mm Hg (with BMI reduction ≥3 kg/m^2^)) [25]
	Waist-height ratio <0.5 is recommended [14]	
Alcohol, smoking, and caffeine	Avoid alcohol intake if possible. Limit daily alcohol intake (2 drinks for men and 1.5 for women, 10g alcohol/drink) [12,14]	According to the GENTSMOKING trial smoking cessation reduced SBP (-6 mm Hg), DBP (-2 mm Hg), mean arterial pressure (MAP: -3 mm Hg), and heart rate (-5 beats/min) [26]
	Avoid smoking. Moderate consumption of coffee is recommended. Energy drinks should be avoided [10,14]	A systematic review and meta-analysis by Cecchini et al. (23 studies) observed that there exists a positive association between increased alcohol intake and hypertension risk [27]
		Meta-analysis conducted by D’Elia et al. (4 studies, n=196256) reported that moderate consumption of coffee is not associated with hypertension risk [28]
		An RCT by Shah et al. and a meta-analysis by Farhangi et al. report that sugar-sweetened beverages increase BP and QTc interval [29,30]
Physical activity	Aerobic exercise: 75-150 minutes/week or 30 minutes on 5-7 days/week [10,12,14]. Examples: walking, jogging, cycling, swimming; brisk walking (50-60 minutes, 3-4 times/week); HIIT [11]	A systematic review and meta-analysis by Hassan et al. (27 studies, n=15220), in children and adolescents, reported that physical activity combined with MNT reduced SBP and DBP [31]
	Dynamic or isometric resistance: 2-3 days per week or 90-150 minutes/week [10,12,14]	30 minutes/week of aerobic exercise reduced SBP (-1.78 mm Hg), DBP (-1.23 mm Hg), and MAP (-1.37 mm Hg), and 150 minutes/week of exercise reduced SBP (-7.23 mm Hg) and DBP (-5.58 mm Hg), according to Ganjeh et al. [32]
		Edwards et al. (270 studies) reported that resting SBP and DBP were reduced in aerobic exercise (-4.49/-2.53 mm Hg), dynamic resistance training (-4.55/-3.04 mm Hg), combined training (-6.04/-2.54 mm Hg), HIIT (-4.08/-2.50 mm Hg) and isometric exercise training (-8.24/-4.00 mm Hg) [33]
Stress reduction, yoga, and meditation	Reduction in chronic stress: yoga and meditation (mindfulness) to be practiced [11,13,14]	According to Ahuja et al. (8 studies, n=482), yoga nidra reduced SBP (WMD=12.03 mm Hg, 95% CI (7.12, 16.93)) and DBP (WMD=6.32 mm Hg, 95% CI (3.53, 9.12)) [34]
		Wu MA et al. (n=3515) reported that yoga moderately reduced SBP and DBP (-0.47/-0.47 mm Hg) [35]
		Mindfulness-based interventions reduced SBP (-9.12, 95% CI (-12.18, -6.05)) and DBP (-5.66, 95% CI (-8.88, -2.43)) [36]

**Table 2:** Consensus recommendation for non-pharmacological management CKD: chronic kidney disease; DASH: dietary approaches to stop hypertension; BP: blood pressure

Consensus recommendations
Prioritize whole grains, vegetables (especially high-nitrate ones like leafy greens and beets), fruits, low-fat dairy, poultry, fish, legumes, nuts, seeds, and unsaturated oils
Aim to reduce sodium intake to less than 1500 mg/day or at least below 1000 mg/day, while avoiding processed and fast foods
Increase potassium intake to 3.5-5 g/day through natural sources, such as bananas and potatoes; however, restrict it in CKD to less than 2.4 g/day
Weight loss through diet (DASH or Mediterranean) and exercise is recommended
Quit smoking, and avoid or limit alcohol intake
Engage in 75-150 minutes of moderate or vigorous exercise weekly, such as brisk walking, jogging, or cycling
Practice yoga or meditation regularly to alleviate chronic stress and reduce BP

*Pharmacological Management of Hypertension* 

While adopting healthy lifestyle habits can reduce BP and lower overall cardiovascular risk, most individuals with hypertension will still require antihypertensive medication alongside these lifestyle changes. Monotherapy is typically preferred for individuals with grade 1 hypertension who have no comorbidities. However, in cases where comorbidities are present or when monotherapy does not adequately control BP, combination therapy is recommended. The benefits of combination therapy include greater BP reduction, lower individual doses, fewer adverse effects, the ability to target multiple physiological pathways affecting BP, enhanced protection for target organs, improved treatment adherence, and reduced overall treatment costs [37,38]. A systematic review and meta-analysis by Wang et al. (7 studies, n=1918) reported that a greater mean reduction in systolic blood pressure (SBP) (-7.4, 95% CI (4.3-10.5)), and a greater proportion of individuals achieved BP <140/90 mm Hg with low-dose combination therapy when compared to monotherapy [39]. 

Guidelines: Antihypertensive treatment is recommended for individuals whose BP exceeds 140/90 mm Hg [12]. According to all major guidelines, first-line therapy should include renin-angiotensin system inhibitors, specifically angiotensin-converting enzyme (ACE) inhibitors and ARBs, alongside thiazide or thiazide-like diuretics and CCBs [10-13]. The European Society of Cardiology (ESC) suggests using second- or third-generation β-blockers for patients with cardiac disorders such as angina, HF, or a history of myocardial infarction, while recommending other classes like α-blockers and mineralocorticoid receptor antagonists (MRAs), such as spironolactone, if initial approaches fail to adequately lower BP [10]. Monotherapy is preferred in cases of initial or high-normal BP and in elderly or frail individuals, while upfront low-dose dual combination therapy, either an ARB or ACE inhibitor with a CCB, or an ARB or ACE inhibitor with a diuretic, is often recommended for more effective control [10,12-14]. To enhance adherence, reduce pill burden, and minimize adverse effects, low-dose single-pill combinations (SPC) or fixed-dose combinations (FDC) are preferred [12].

Monotherapy: According to a systematic review by Pinakesty et al., ARBs (telmisartan) monotherapy produced significant BP reductions at both single-dose (5.61/5.15 mm Hg) and double-dose regimens (21.8±5.59/16.00±5.97 mm Hg) [40]. A meta-analysis by Kumar et al., which pooled data from 15 studies involving 1926 participants, confirmed ARBs (telmisartan) efficacy in lowering SBP by a weighted mean difference (WMD) of 2.69 mm Hg (95% CI: 1.38-4.00) and diastolic blood pressure (DBP) by 1.26 mm Hg (95% CI: 0.45-2.08) [41]. In an RCT by McDermott et al., patients with peripheral artery disease experienced decreases in SBP from 135.58±14.98 mm Hg to 128.93±17.72 mm Hg, in DBP from 74.24±9.22 mm Hg to 69.06 mm Hg, and in mean arterial pressure from 94.68±9.31 mm Hg to 89.01±12.29 mm Hg after six months of ARB treatment [42]. Regarding CCBs (cilnidipine), Harlalka et al. demonstrated that the therapy led to significant reductions in SBP (142.75±9.17 to 131.5±7.92 mm Hg), DBP (87.88±8.97 to 79.63±7.33 mm Hg), and mean BP (MBP: 106.16±3.89 to 96.92±3.32 mm Hg) over 12 weeks [43]. However, an RCT by Jj et al. found that CCBs did not significantly affect SBP and DBP but did reduce pedal edema and heart rate by 1.16 beats/min [44]. In a meta-analysis covering 24 studies, the pooled between-group effect sizes showed no statistically significant difference between cilnidipine and control therapy for change in SBP (Hedge’s g=0.05; 95% CI: -0.13 to 0.24; p=0.573) or DBP (Hedge’s g=0.66; 95% CI: -0.18 to 1.50; p=0.122) [45]. Sawant et al., in the START ABPM study, randomized forty subjects to ARBs (telmisartan 40 mg) or CCBs (cilnidipine 10 mg) groups and observed reductions in 24-hour, six-hour, and segmental SBP/DBP across both drugs, with telmisartan providing superior sustained 24-hour BP control, especially in the early morning period [46]. Furthermore, an RCT by Sayeli et al. involving 100 participants revealed that after twelve weeks, mean SBP reduction with CCBs (22.7±8.2 mm Hg) exceeded that of ARBs (18.9±7.5 mm Hg), while reductions in DBP were comparable (CCBs: 12.3±5.2 mm Hg; ARBs: 11.7±4.8 mm Hg) [47]. 

Combination therapy: An RCT by Ram et al. reported that individuals receiving combination therapy with an ARB and a CCB (telmisartan 40 mg and cilnidipine 10 mg) showed significant reductions at the end of eight weeks in mean BP (144.5±10.2 to 123.0±10.0 mmHg; p<0.001), office SBP (144.5±10.2 to 123.0±10.0 mmHg; p<0.0001), office DBP (91.4±8.0 to 81.3±9.0 mmHg; p<0.0001), and aortic augmentation index (27.5±4.6 to 22.3±12.2; p=0.0178) [48].

Management of Hypertension With Comorbidities

Hypertension frequently coexists with other chronic conditions, including CVD, chronic kidney disease (CKD), type 2 diabetes mellitus (T2DM), and obesity. Effective management necessitates a comprehensive approach that targets both hypertension and these coexisting conditions, aiming to reduce overall risk and enhance patient outcomes. HMOD can manifest even in individuals with less severe hypertension, including asymptomatic individuals with moderately elevated BP, as well as those with long-standing or severe hypertension. Guidelines emphasize that an evaluation for HMOD should be conducted as soon as a hypertension diagnosis is confirmed [13]. According to a pan-India survey, cilnidipine along with telmisartan is useful in individuals with uncontrolled hypertension, diabetes, and CKD [49].

*Cardiovascular Disorders* 

High SBP is the most common modifiable risk factor for CVD and a major cause of mortality globally [50]. The Longitudinal Ageing Study in India found that CVD prevalence was notably higher among individuals with hypertension (7.4% in women and 8.8% in men) compared with those without hypertension (1.9% in women and 3.5% in men). Individuals with hypertension had approximately twice the odds of developing CVD compared with their non-hypertensive counterparts (adjusted odds ratios (aORs): 2.60, 95% CI: 2.08-3.25 for women and 1.88, 95% CI: 1.54-2.29 for men) [51]. Refer to Table 3 for drug preferences [6,8,10].

**Table 3:** Recommended drugs for management of hypertension and comorbidities ACEi: angiotensin-converting enzyme inhibitor; ARB: angiotensin II receptor blocker; BB: beta blocker; CCB: calcium channel blocker; MRA: mineralocorticoid receptor antagonist; SGLT-2 inhibitors: sodium-glucose cotransporter-2 inhibitors; DHP: dihydropyridine calcium channel blocker; GLP-1 RA: glucagon-like peptide-1 receptor agonist; HF: heart failure; CKD: chronic kidney disease; T2DM: type 2 diabetes mellitus; ARNI: angiotensin receptor-neprilysin inhibitor

Comorbidity	Recommended drug
Coronary artery disease	ACEi/ARB+BB/CCB (symptomatic angina: BB/CCB)
HF	ARNI/ACEi/ARB+BB+diuretics+MRA+SGLT-2 inhibitors; avoid: non-DHP CCB
Stroke	ACEi/ARB+CCB/diuretics (thiazide or thiazide-like); lipid-lowering therapy; antiplatelet therapy for ischemic stroke
Aortic valve stenosis/valvular heart disease	ACEi/ARB+BB
Atrial fibrillation	ACEi/ARB+CCB/diuretic
CKD	ACEi/ARB+CCB/diuretics (monitor kidney function and electrolytes) SGLT-2 inhibitors can also be used
T2DM	ACEi/ARB+CCB/diuretic SGLT-2 inhibitors and GLP-1 RA preferred; avoid BB
Chronic obstructive pulmonary disease	ACEi/ARB+CCB

Guidelines: Risk assessment for CVD should be performed using tools such as SCORED-2 and SCORED-OP [10]. In adults with a history of CVD, primarily coronary artery disease (CAD), drug treatment should be initiated at a BP range of ≥130/80 mm Hg according to some guidelines, while the ISH and InSH recommend starting treatment at >140/90 mm Hg [10,12-14]. Regarding target BP, the ESC suggests a goal of <120-129/70-79 mm Hg if tolerated; in cases where this is not well tolerated, it is advised to target BP that is as low as reasonably achievable. In contrast, other guidelines recommend a target of <130/80 mm Hg [10,12,14]. For elderly individuals, the ISH advises a target BP of less than 140/80 mm Hg [14]. In patients with HF, the recommended BP target is below 130/80 mm Hg but above 120/70 mm Hg [14]. Both the ESC and InSH recommend the use of sodium-glucose cotransporter-2 (SGLT-2) inhibitors for HF management and caution against the use of nondihydropyridine CCBs in this population [10,12-14].

Evidence: A meta-analysis by Escobar et al., which included 16 studies, found that ACEi and ARBs have a similar impact on clinical outcomes in individuals with myocardial infarction. The risk ratios (RR) for major adverse cardiovascular events (MACE) (RR: 1.03, 95% CI: 0.88-1.20), all-cause mortality (RR: 1.03, 95% CI: 0.88-1.20), cardiovascular mortality (RR: 1.00, 95% CI: 0.89-1.12), stroke (RR: 1.03, 95% CI: 0.80-1.32), and hospitalization due to HF (RR: 0.99, 95% CI: 0.90-1.09) were all comparable between the two drug classes [52]. In a systematic review and meta-analysis by Wanas et al., which included 45 studies with a total of 170794 participants, ARBs were not associated with increased risk of all-cause mortality (RR: 1.00; 95% CI: 0.97-1.04), myocardial infarction (RR: 1.01; 95% CI: 0.96-1.06), or stroke (RR: 0.92; 95% CI: 0.83-1.01), indicating their cardiovascular safety profile [53]. Manal et al. conducted a larger systematic review and meta-analysis of 72 studies involving 297451 participants and reported that both ACE inhibitors and ARBs reduced the risk of stroke. ACEi reduced stroke risk by 14% (RR: 0.86; 95% CI: 0.76-0.98; p=0.02), while ARBs reduced it by 9% (RR: 0.91; 95% CI: 0.86-0.97; p=0.003) [54]. According to a systematic review and meta-analysis, ARBs reduced both hospitalization due to HF (RR: 0.89) and worsening of HF (RR: 0.91) [55]. Furthermore, the TEDY trial demonstrated that telmisartan effectively reduced peak wall stress (PWS) by -4.19 kPa/year (95% CI: -8.24 to -0.14; p=0.043) and peak wall rupture index (PWRI) by -0.014 (95% CI: -0.026 to -0.001; p=0.032) in individuals with aortic aneurysm, likely through its BP-lowering effects [56]. 

Chronic Kidney Disease

Diabetes and hypertension are the leading causes of CKD worldwide. Based on various studies, more than 85% of individuals with CKD have hypertension [57-60]. According to an Indian study, the prevalence of CKD in individuals with hypertension is 14% [61]. The RR for incident CKD or end-stage renal disease (ESRD) associated with hypertension vs. ideal BP was 1.56 (95% CI, 1.39-1.75) in women and 2.06 (95% CI, 1.64-2.60) in men [62]. According to an Indian survey, clinicians suggested combining cilnidipine with telmisartan for enhanced cardiovascular and renal protection [63]. Refer to Table 3 for drug preferences. 

Guidelines: Individuals should be screened for CKD at the time of diagnosis of hypertension and reviewed annually [11,22]. For individuals with mild CKD and elevated BP, a cardiovascular risk assessment should be performed prior to initiating BP-lowering therapy. For individuals with or without diabetes who have moderate to severe CKD and BP ≥140/80 mm Hg (or ≥130/80 mm Hg according to the ESC), lifestyle modifications along with BP-lowering medications are recommended [10,14]. The InSH guidelines specifically recommend initiating therapy when BP exceeds 140/90 mm Hg [14]. Target BP goals vary slightly among guidelines: the Kidney Disease: Improving Global Outcomes (KDIGO) guidelines recommend SBP of less than 120 mm Hg (if tolerated), while for individuals with an estimated glomerular filtration rate (eGFR) greater than 30 mL/min/1.73 m², the ESC suggests an SBP target between 120-129 mm Hg [10,64]. Typically, treatment involves a combination of an ACEi or an ARB, both of which reduce albuminuria, combined with a CCB or a diuretic. SGLT-2 inhibitors are recommended for individuals with an eGFR above 20 mL/min/1.73 m², depending on specific drug indications [10]. Additionally, SPCs containing cilnidipine (10 mg) are advised for lowering BP in patients with microalbuminuria or proteinuria, sympathetic overactivity, and pedal edema [11]. 

Evidence: A meta-analysis by Kumari et al., which included seven studies with a total of 289 participants, reported that CCBs (cilnidipine) effectively reduced SBP by a WMD of 4.33 mm Hg (95% CI: 1.26 to 7.31) and proteinuria by a WMD of 0.61 (95% CI: 0.42 to 0.80) in individuals with CKD and hypertension [65]. An RCT by Ito et al. involving patients undergoing chronic hemodialysis demonstrated that CCBs significantly lowered post-dialytic SBP and notably decreased the rise in SBP during dialysis sessions, from 12.0±15.4 mm Hg to 4.8±10.1 mm Hg [66]. According to a meta-analysis by Srivathsan et al. encompassing 11 studies, individuals treated with CCBs (cilnidipine) showed improvements in serum creatinine (standardized mean difference (SMD): -0.022; 95% CI: -0.143 to 0.0987), urinary protein excretion and urinary protein-to-creatinine ratio (UPCR), and eGFR (SMD: 0.693; 95% CI: values as reported) [67]. Additionally, an RCT by Harlalka et al. reported that CCBs significantly reduced the urinary albumin-to-creatinine ratio (UACR) from 152.82±82.39 to 39.55±17.28 mg/g in hypertensive individuals after 12 weeks of treatment [43]. The JINNAGA trial found that ARBs (telmisartan 80 mg) reduced proteinuria from 0.51±0.60 to 0.27±0.36 in patients with CKD [68].

*Diabetes* 

Individuals with T2DM are three times more likely to develop hypertension compared to those without diabetes. Approximately 50%-80% of individuals with T2DM have elevated BP [69]. The simultaneous occurrence of these two chronic conditions poses a significant healthcare challenge, as it increases the risk of cardiovascular complications and accelerates the progression of kidney disease [70]. A study in India found that the prevalence of hypertension was 58% among individuals who self-reported T2DM, 41% among those diagnosed with T2DM during the study, and 35.8% in individuals identified as high-risk for developing T2DM [71]. According to a clinician's survey, two-thirds of clinicians reported that the cilnidipine-telmisartan combination provides benefits beyond BP reduction, including cardioprotection, vasoprotection, and improved glycemic control, and 76% specifically recommended this combination for the management of hypertension in patients with diabetes [72]. Refer to Table 3 for drug preferences. 

Guidelines: Risk estimation with the use of SCORED2-diabetes should be considered [10]. Lifestyle modifications alongside BP-lowering medications are recommended for individuals with BP ≥140/80 mm Hg, or ≥130/80 mm Hg according to the ESC and the ACC/AHA [10,12,14]. The InSH and the ISH guidelines recommend initiating therapy for those with BP >140/90 mm Hg [13,14]. Target BP goals are set at 120-129/70-79 mm Hg by ESC, and 130/80 mm Hg by the ADA Standards of Care 2024, ISH, InSH, and ACC/AHA [10,12-14,73]. Typically, individuals require a combination of ACEi or ARB, along with a CCB or diuretics [10,11]. Among CCBs, cilnidipine is considered a more effective and safer option for individuals with diabetes and hypertension in India, especially when combined with ARBs [11,70]. Among ARBs, telmisartan and losartan are the most preferred choices. ACEi or ARBs can also be used in combination with SGLT-2 inhibitors for managing diabetes and hypertension [11]. 

Evidence: CCBs (cilnidipine) significantly reduced mean SBP from 150.07±5.44 to 123.03±5.23 mm Hg and microalbuminuria from 66.62±8.39 to 38.8±6.45 mg/L after six months in individuals with mild to moderate hypertension and diabetes [74]. A systematic review and meta-analysis of seven studies involving 1757 participants found that combination therapy with SGLT-2 inhibitors and ACEi or ARBs resulted in reductions in SBP (WMD: -3.84 mm Hg), DBP (WMD: -2.08 mm Hg), 24-hour ambulatory SBP (WMD: -4.59 mm Hg), 24-hour ambulatory DBP (WMD: -2.08 mm Hg), UACR (WMD: -29.70%), eGFR (WMD: -3.46 mL/min/1.73 m²), HbA1c (SMD: 0.48), fasting plasma glucose (SMD: -0.28), uric acid (SMD: -0.35), and body weight (SMD: -0.29) [75]. In an RCT conducted by Lee et al., at 24 weeks, fasting glucose levels significantly decreased from 111.2±10.2 mg/dL to 107.7±13.4 mg/dL (p=0.039), and insulin resistance measured by HOMA-IR was significantly lower in the telmisartan-statin group compared to controls [76]. Table 4 shows the consensus recommendation for the management of hypertension and comorbidities.

**Table 4:** Consensus recommendation for the management of hypertension and comorbidities CVD: cardiovascular disorders; T2DM: type 2 diabetes mellitus; CKD: chronic kidney disease; SBP: systolic blood pressure; ACE: angiotensin-converting enzyme; ARB: angiotensin II receptor blocker; CCB: calcium channel blocker; BP: blood pressure; HF: heart failure; SGLT-2: sodium-glucose cotransporter-2; SPC: single-pill combination; eGFR: estimated glomerular filtration rate; GLP-1: glucagon-like peptide-1; RAS: renin-angiotensin system

Pharmacological management of hypertension
Pharmacological therapy is recommended for most adults with BP ≥140/90 mm Hg; in individuals with BP in the range of 130-139/80-89 mm Hg, initiation of pharmacological treatment should be considered when there is the presence of established CVD, T2DM, CKD, or a high global cardiovascular risk. Target BP: <130/80 mm Hg (if tolerated); elderly (SBP 130-139 mm Hg)
RAS inhibitors (ACE inhibitors and ARBs), along with thiazide or thiazide-like diuretics, and CCBs, are the first-line options for managing hypertension
Prescribe β-blockers specifically in patients with coexisting cardiac conditions such as angina, HF, or a history of myocardial infarction
ARB-CCB combinations are recommended as they provide greater BP reduction, improved tolerability, a lower pill burden that enhances adherence, and additional organ-protective benefits beyond BP control
Hypertension and CVD
CVD risk assessment should be performed for appropriate risk stratification. In individuals with established CVD, antihypertensive therapy should be initiated at a BP of ≥130/80 mm Hg. Target: <130/80 mm Hg
Preferred pharmacological treatment includes an ACEi or an ARB in combination with a CCB or a β-blocker, selected according to the specific clinical indication
Special considerations include avoiding non-dihydropyridine CCBs in patients with HF and incorporating SGLT-2 inhibitors in the management of HF where clinically indicated
Hypertension and CKD
All individuals should be evaluated for CKD at the time of hypertension diagnosis and reviewed annually thereafter
Antihypertensive therapy should be initiated at a BP threshold of ≥130/80 mm Hg in individuals with CKD or the presence of albuminuria. Target BP: <130/80 mm Hg or SBP <120 mm Hg (if tolerated)
ACEi/ARBs are the first-choice drugs. CCBs or diuretics for additional BP control
SPCs containing cilnidipine (10 mg) are particularly effective in lowering BP, managing microalbuminuria/proteinuria, and addressing sympathetic overactivity and pedal edema
SGLT-2 inhibitors should be considered in eligible individuals with an eGFR ≥20 mL/min/1.73 m²
Hypertension and T2DM
Antihypertensive therapy should be initiated at a BP threshold of ≥130/80 mm Hg. Target: <130/80 mm Hg
Preferred treatment consists of an ACEi/ARB in combination with a CCB or a diuretic
Cilnidipine is considered more effective and safer among CCBs, particularly in combination with ARBs (preferably telmisartan) for individuals with diabetes and hypertension
Where appropriate, therapy should be combined with SGLT-2 inhibitors or GLP-1 receptor agonists to address coexisting metabolic and cardiovascular risk

Side-Effects, Adherence to Therapy and Follow-Up

While ARB-CCB combinations are generally well tolerated and effective for BP control, awareness of potential adverse effects is important. CCBs may cause peripheral edema, dizziness, flushing, and palpitations due to arteriolar vasodilation, although newer agents such as cilnidipine are associated with a lower incidence of these effects [77]. ARBs, including telmisartan, are generally well tolerated but may cause hyperkalemia, hypotension, dizziness, and, rarely, renal impairment due to reduced aldosterone-mediated potassium excretion [78]. Accordingly, periodic monitoring of serum potassium and renal function is recommended, especially in high-risk patients. ARB-CCB combinations can mitigate CCB-related adverse effects, particularly peripheral edema, through ARB-mediated postcapillary venodilation, allowing effective BP reduction with improved tolerability and lower required doses [79].

Adherence to antihypertensive therapy is a critical component in managing hypertension effectively, yet it remains a significant challenge. Factors contributing to poor adherence include the complexity of treatment regimens, poor access to care, side effects of medications, lack of patient education, socioeconomic barriers, and the asymptomatic nature of hypertension, which can lead individuals to underestimate the importance of continued treatment [80,81]. According to a systematic review by Agarwal et al. [82] (49 studies, n=15577), the reported non-adherence rate in India was 54.0% (range 4%-98.5%). Strategies to improve adherence include simplifying treatment through FDCs (SPCs); identifying drug-related adverse effects and appropriate dosing levels; enhancing patient education about the risks of uncontrolled hypertension; using mobile health technology for reminders; prescribing long-acting, once-daily dosing regimens; home BP monitoring; enlisting the support of family members for medication adherence; multidisciplinary care; and regular follow-ups with healthcare providers [10,14]. 

Follow-up visits are essential for effective hypertension management, as they help ensure that BP remains within the target range and that treatment plans are adjusted as needed. Regular follow-up appointments allow healthcare providers to monitor BP levels, assess the effectiveness of current therapies, evaluate lifestyle and medication adherence, and make necessary adjustments. Another crucial aspect of follow-up care is its role in monitoring HMOD [12,81]. Refer to Table 5 for follow-up recommendations [12,13,83]. 

**Table 5:** Follow-up for hypertension BP: blood pressure; HMOD: hypertension-mediated organ damage

Individuals with normal BP should be evaluated annually
Individuals with elevated or stage 1 hypertension with a 10-year cardiovascular risk <10% should be treated with non-pharmacological therapy and have a follow-up within 3-6 months
Individuals with stage 1 hypertension (with a 10-year cardiovascular risk >10%) and those with stage 2 hypertension should be treated with pharmacological therapy and followed up monthly until the target BP is reached, and every 3-6 months thereafter
Individuals without preexisting HMOD can be evaluated every 3 years. Individuals with preexisting HMOD should undergo more frequent evaluations, depending on the type and severity of HMOD

## Conclusions

Hypertension is a major public health concern globally and in India, with significant gaps in diagnosis and control rates. The consensus stresses the importance of managing hypertension beyond just BP control, aiming to reduce complications and improve cardiovascular health outcomes. It underscores the significance of addressing comorbidities such as CVD, kidney disease, diabetes, and other associated conditions. Non-pharmacological interventions, including lifestyle modifications such as a healthy diet, regular physical activity, weight reduction, smoking cessation, stress management, adequate sleep, and reduced intake of alcohol and caffeine, play a crucial role in managing hypertension. The consensus highlights the therapeutic benefits of antihypertensive agents such as telmisartan (ARB) and cilnidipine (CCB), which offer benefits beyond hypertension control, particularly for individuals with cardiovascular and renal complications. These medications are crucial in optimizing long-term patient outcomes.
